# An Update on the HIV DNA Vaccine Strategy

**DOI:** 10.3390/vaccines9060605

**Published:** 2021-06-05

**Authors:** Joseph Hokello, Adhikarimayum Lakhikumar Sharma, Mudit Tyagi

**Affiliations:** 1 Department of Microbiology and Immunology, Faculty of Biomedical Sciences, Kampala International University-Western Campus, P.O. Box 71, Bushenyi 0256, Uganda; hokello.joseph@kiu.ac.ug; 2 Center for Translational Medicine, Thomas Jefferson University, 1020 Locust Street, Philadelphia, PA 19107, USA; LakhikumarSharma.Adhikarimayum@jefferson.edu

**Keywords:** lentiviral vectors, DNA vaccines, immunization, HIV, AIDS

## Abstract

In 2020, the global prevalence of human immunodeficiency virus (HIV) infection was estimated to be 38 million, and a total of 690,000 people died from acquired immunodeficiency syndrome (AIDS)–related complications. Notably, around 12.6 million people living with HIIV/AIDS did not have access to life-saving treatment. The advent of the highly active antiretroviral therapy (HAART) in the mid-1990s remarkably enhanced the life expectancy of people living with HIV/AIDS as a result of improved immune functions. However, HAART has several drawbacks, especially when it is not used properly, including a high risk for the development of drug resistance, as well as undesirable side effects such as lipodystrophy and endocrine dysfunctions, which result in HAART intolerability. HAART is also not curative. Furthermore, new HIV infections continue to occur globally at a high rate, with an estimated 1.7 million new infections occurring in 2018 alone. Therefore, there is still an urgent need for an affordable, effective, and readily available preventive vaccine against HIV/AIDS. Despite this urgent need, however, progress toward an effective HIV vaccine has been modest over the last four decades. Reasons for this slow progress are mainly associated with the unique aspects of HIV itself and its ability to rapidly mutate, targeting immune cells and escape host immune responses. Several approaches to an HIV vaccine have been undertaken. However, this review will mainly discuss progress made, including the pre-clinical and clinical trials involving vector-based HIV DNA vaccines and the use of integrating lentiviral vectors in HIV vaccine development. We concluded by recommending particularly the use of integrase-defective lentiviral vectors, owing to their safety profiles, as one of the promising vectors in HIV DNA vaccine strategies both for prophylactic and therapeutic HIV vaccines.

## 1. Introduction

In the 1990s, the concept of DNA vaccination was introduced following the observation that an intramuscular injection with naked DNA triggered the expression of coded antigens [1]. Subsequently, Tang et al. [2] demonstrated that this approach elicited an immune response against the expressed antigen. Much interest in DNA vaccines was generated when it was discovered that the immune response induced following DNA injection was strong enough to protect mice and chicken against a challenge with an experimental influenza virus [3,4].

Indeed, unlike naked DNA vaccines, vector-based DNA immunization is a promising new approach to prevent those infectious diseases, for which classical vaccines, consisting of attenuated or inactivated pathogens or, more recently, recombinant proteins, do not have positive effects. Given that the live-attenuated or inactivated vaccines have been successfully used for several diseases [5], there are still diseases for which the use of live-attenuated pathogens could be problematic and of very high risk. Notably, these kinds of vaccines could be injurious to individuals with compromised immune systems, including cancer patients undergoing chemotherapy, AIDS patients, newborns, or the elderly. Moreover, there is a real danger of the live attenuated viruses reverting into virulence through mutations. In the case of HIV/AIDS, the risk of reversion to virulence could be fatal, and thus, unacceptable.

Interestingly, vector-based DNA vaccines also elicit durable and strong cell-mediated and humoral immune responses without any of the risks associated with live attenuated vaccines [6].

Recent HIV DNA vaccine research efforts have mainly focused on utilizing HIV-based lentiviral vectors for antigen delivery. Despite concerns pertaining to the safety of HIV-based lentiviral vectors for vaccine delivery and gene transfer, HIV DNA vaccination strategy presents several advantages as discussed below. Notably, in addition to their use for transducing HIV-specific target cells or for in vivo gene therapy for HIV/AIDS infection, the lentiviral vectors, in particular, can be pseudotyped into a recombinant viral form that can infect different target cells, including neurological and cancer cells.

Generally, DNA vaccines can be delivered through any of the following different routes: intramuscular [7], intradermal [7], subcutaneous [8], oral [9], intranasal [10,11,12,13,14,15], intraperitoneal [7], intravenous [7], and vaginal [16,17]. Usually, needle injection via intramuscular and intradermal routes is the most effective way to deliver vector-based DNA vaccines. However, recently an alternative and very efficient method for intradermal delivery was established. This method includes bombarding the target cells with DNA-coated microparticles using a “gene gun”. Usually, inert gold microparticles covered with specific recombinant DNA sequences are used as vaccine, and DNA-coated gold particles are shot through the skin with gas pressure, normally helium [18,19].

The capacity of vector-based DNA vaccines in inducing both cell-mediated and humoral immune responses is the most crucial feature of this strategy of vaccination [20,21,22]. This characteristic makes vector-based DNA vaccination the most appropriate strategy for the prevention of diseases including HIV/AIDS [23].

## 2. Recent Animal and Human Studies of Different Vector-Based HIV DNA Vaccine Candidates

Some of the main limitations of using plasmid DNA as a vaccine for HIV are: (i) inability to deliver the DNA efficiently and (ii) low expression of plasmid DNA. Therefore, efforts have been made to circumvent these limitations, so that DNA viral vectors can be exploited for use in HIV vaccines. An efficient HIV vaccine should be able to elicit strong humoral and cellular immune responses, including the CD4+ and CD8+ T-cell responses. One novel approach comprises priming with DNA vaccine and boosting with HIV envelope (HIV-Env) or virus-like particles. To this effect, several animal and human trials have been conducted using vector-based HIV DNA vaccines and the results of these trials have been very promising. A summary of the advantages and disadvantages of the different viral vectors for DNA vaccine delivery is provided in Table 1. However, for a detailed review of these vectors, please refer to Vannucci et al. [24] and Ura et al. [25].

Flatz et al. **[34]** demonstrated earlier that prime-boost vaccination with mismatched simian immunodeficiency virus (SIV) envelope (Env) gene derived from SIVmac239 prevented intrarectal infection by SIVsm660. In this case, analysis of different gene-based, prime-boost immunization regimens showed that recombinant adenovirus type 5 (rAd5)–prime followed by replication-deficient lymphocytic choriomeningitis virus (rLCMV)–boost elicited robust CD4+ and CD8+ T-cells and humoral immune responses. Subsequently, Shen et al. [35] examined if the addition of a glycoprotein 120 (gp120) protein in alum or Modified Vaccinia Ankara (MVA)–expressed secreted gp140 (MVAgp140) augments the immunogenicity of a DNA prime–MVA boost vaccine in rhesus macaques. In this case, they observed that both boost immunogens enhanced the breadth of HIV-1gp20 and variable regions V1V2 antibody responses. Interestingly, the gp120 boost elicited earlier and robust responses while the MVAgp140 resulted in improved antibody durability. In Thailand, Rerks-Ngarm et al. [31] evaluated four priming injections using recombinant canarypox vector vaccine (ALVAC-HIV) plus two booster injections of a recombinant gp120 subunit vaccine (AIDSVAX B/E). Results of this trial demonstrated that ALVAC-HIV and AIDSVAX B/E vaccine-elicited vaccine-induced HIV-1 Env V1V2–directed antibodies, though this vaccine exhibited modest vaccine efficacy of 31.2%. In another development, in a phase I clinical trial in which plasmid DNA vaccines encoding HIV antigens were administered, results demonstrated that there were low CD4+ and CD8+ T-cell responses in the absence of adjuvants and boosting with the alternative vaccine. The authors further observed that multiple factors, including both the dose and number of vaccinations, affect the immunogenicity of plasmid DNA vaccines in human clinical trials [36].

Furthermore, Nilsson et al. [37] compared the safety and the immunogenicity of intradermal vaccination with or without electroporation in a phase I, randomized, placebo-controlled trial of HIV-DNA-prime and HIV-MVA-boost vaccine in healthy Swedish volunteers. They found that intradermal or electroporation delivery was well tolerated and that, following three HIV-DNA immunizations, there were no statistically significant differences in interferon-gamma (IFN-γ) response to HIV-Gag between HIV-DNA intradermal and electroporation recipients and HIV-DNA intradermal recipients. Usually, immunization regimens that have been assessed for the development of HIV DNA vaccines have utilized purified HIV-Env proteins for boosting components of the vaccine regimen. However, Pantaleo et al. [38], for the first time, recently implored the effects of co-administration of HIV-Env proteins with either DNA or NYVAC vectors during the priming to determine whether it would result in early antibody response to HIV-Env V1V2 regions. Interestingly, they observed that co-administration of HIV gp120 Env protein together with DNA or NYVAC vectors during priming led to an early and more potent induction of Env V1V2 IgG antibody responses, suggesting that this immunization approach should be considered for induction of preventive antibodies in future HIV vaccine efficacy trials.

## 3. Lentiviral Vector Approach for HIV DNA Vaccines and Dendritic Cell Targeting

The HIV DNA vaccines research mainly utilizes vector-based antigen delivery approaches primarily including adenoviral and pox vectors [39,40,41,42,43,44,45,46]. Although additional viral vectors (Table 1), especially those derived from cytomegalovirus and HIV-based lentiviral constructs were found promising in pre-clinical studies, the safety concerns impede their progress [47,48,49,50]. In this review, we particularly emphasize on the recent advances in the design and development of HIV-based lentiviral vectors for HIV vaccines that enhance adaptive immune responses.

Lentiviral vectors are ideal vehicles for the deliveries of transgenes because of their ability to integrate into the host cell genome and maintain persistent gene expression. In addition, lentiviral vectors can transduce cells that are in the mitotic and post-mitotic stages of the cell cycle, and therefore they offer the opportunity to target both the dividing and non-dividing cells [51]. Although lentiviral vectors have been extensively explored for gene therapy applications [52,53], their use as prophylactic HIV vaccines is considerably less developed, though pre-clinical studies have shown promise [48,49,54,55,56,57]. The ability to integrate into both dividing and non-dividing cells makes lentiviral vectors efficient vehicles to deliver therapeutic genes with sustained expression, meaning that the therapeutic effects of the transgenes can be long-lasting, although this can also be a safety issue. The major distinction between these two applications lies for the most part in the genes that are delivered and desired target cells.

Lentiviral vector–based anti-HIV gene therapies that primarily target hematopoietic stem cells (HPSCs) or T cells confer host resistance to infection through the delivery of genetic information. This results in the induction of specific immune defense that interferes with the HIV life cycle, including entry or replication. However, lentiviral vaccines for HIV that target antigen-presenting cells (APCs) for efficient HIV antigen delivery to immune cells promote the presentation of antigens along with the major histocompatibility complex (MHC) molecules for better immune response. In this regard, dendritic cells (DCs) are the most suitable prophylactic vaccine targets because DCs are the most efficient APCs and are able to trigger both a strong and long-lasting antigen-specific T-cell responses [58,59]. The use of lentiviral vectors as HIV DNA vaccines targeting DCs offers several advantages, including: (1) continuous antigen production following integration into the host cell genome, (2) endogenous production of antigen with all required post-translational modifications for a well-tailored MHC presentation, (3) the ability to encode immunostimulatory genes and checkpoint inhibitors to enhance T-cell responses, and (4) minimal antigenicity when using certain pseudo-typed constructs such as the lentiviral vectors pseudo-typed with vesicular stomatitis virus protein G (VSV-G) [48,57].

The advantage of DC-based vaccines is that DCs play an important role in initiating and regulating innate and adaptive responses with the unique ability to activate both the naïve CD4+ and CD8+ T cells. Initially, HIV-1-based lentiviral vectors were pseudo-typed with VSV-G glycoprotein, which allowed the generation of the highly infectious virus with a broad tropism for target cell transduction [60]. Subsequently, for minimizing the off-target effects, the use of other glycoproteins for pseudo-typing was explored to enhance safety and specificity, for example, lentiviral vectors pseudo-typed with a mutated Sindbis virus glycoprotein (SVGmu). This improvement greatly enhances the vector tropism toward human DCs because SVGmu selectively binds to DC-SIGN or CD209, the two abundant DC surface proteins [61]. The standard laboratory-adapted Sindbis virus envelopes, which besides targeting DC-SIGN, also target ubiquitously expressed heparan sulfate proteoglycans on cell surfaces. However, SVGmu contains mutations in the heparan sulfate binding site, which abolish its binding and heparan sulfate–mediated cell entry. Interestingly, it was found that a single injection of an SVGmu-pseudotyped lentiviral vector expressing HIV-1 Gag in mice was able to activate their DCs and promote a durable HIV-1-specific immune response, while minimal vector immunity was observed [61,62]. Of note, the prime/boost regimens consisting of either a heterologous DNA prime/SVGmu-Gag boost or successive SVGmu-Gag injections enhanced both humoral and cellular responses and was found to perform better than a DNA prime/adenoviral vector boost immunization in terms of both the breadth and polyfunctionality of the vaccine-induced Gag-specific CD8+ and CD4+ T-cells. Moreover, the specificity of SVGmu toward DC-SIGN was further enhanced when amino acid substitutions in the receptor-binding site and wild-type furin cleavage site were restored in the SVGmu. These changes augmented the proteolytic processing of SVGmu and virus maturation, which led to enhanced specificity for DC-SIGN [63].

Based on the approach of using DC-specific glycoprotein, SVGmu, Bryson et al. [64] and Lee et al. [65] developed producer cell lines that allow for high titer production of lentiviral particles based on concatemeric DNA transfection as opposed to transient transfection. Ex vivo differentiation of conventional DCs capable of stimulating naïve T cells for potential immunotherapeutic applications is complex, requiring several days to complete. In addition, clinical trials have proven poor trafficking of conventional DCs from subcutaneous injection sites to lymph nodes where DCs stimulate naïve T cells for long-lasting memory response. However, to overcome these problems, Stripecke [66] demonstrated that an overnight ex vivo lentiviral gene transfer into DC precursors for the production of cytokine combinations and antigens was sufficient to induce autonomous self-differentiating, antigen-loaded DCs both in vitro and in vivo. The induced DCs efficiently migrated from the skin injection sites to the lymph nodes where they effectively activated de novo antigen-specific effector memory T cells. Similarly, using integrase-defective lentiviral vectors (IDLVs), Daenthanasanmak et al. [67] demonstrated that IDLVs expressing combinations of cytokines (GM-CSF/IL-4) or GM-CSF/IFN-α) that are used to transduce human monocytes generated functional DCs. In a related development, Cousin et al. [68] demonstrated strong and persistent, specific cytotoxic T lymphocyte (CTL) responses induced by IDLVs which persisted for several months following a single injection. Furthermore, they observed that the CTL responses were associated with the induction and maturation of DCs.

To further improve the design and provide specificity to lentiviral vectors for DC targeting, the vectors were pseudotyped with measles virus glycoproteins (MVGs), hemagglutinin (H), and fusion (F). Given that MVG-pseudotyped lentiviruses fuse at the plasma membrane for direct cell entry, it offered an added advantage to MVGs over SVGmu and VSV-G-pseudo-typed lentiviruses, which require endocytosis for viral membrane fusion and cell entry [69]. The H glycoprotein of the measles virus selectively binds to both the CD46 receptor, which expressed on all the nucleated cells and the signaling lymphocyte activation molecule (SLAM) receptor that constitutively expressed on DCs, thymocytes, memory T-cells, B-cells, and monocytes [70]. Hence, measles virus pseudo-typed lentivectors showed four-fold more infection capability in DCs than VSV-G-pseudo-typed vectors [71]. Another advantage of MVGs-pseudotyped lentiviruses is that they do not affect the maturation and activation status of the transduced DCs; thus unintended DC stimulation is minimal. On the contrary, VSV-G-pseudo-typed lentiviruses are known to stimulate transduced DCs.

To further improve MVGs-pseudotyped lentiviral vectors specificity for APCs, mutations were made in the H glycoprotein’s motifs responsible for CD46 and SLAM binding, and the protein was further modified to display single-chain antibodies (scFv) directed against class II MHC [72,73,74]. The resultant lentiviral vectors were found to have high in vivo DC specificity and significantly enhanced CD4+ and CD8+ T cell responses; however, the responses were still less than VSV-G-pseudotyped vectors, probably due to the impaired transduction efficiency and loss of the chimeric constructs. However, mice immunized with a single injection of the HIV-1-derived lentiviral vector pseudo-typed with MHCII-targeted MVGs elicited antigen-specific effector CD4+ and CD8+ T cells and established T-cell immune memory, showing their potential for clinical or vaccine use [73].

Another strategy took the benefit of the budding process of viral particles and the inclusion of a cell-targeting moiety at the surface of viral particles. The lentiviral vectors were pseudo-typed with a binding-defective, fusion-competent VSV-G glycoprotein and with DC-specific single variable regions derived from camel IgG sequences called nanobodies. The resultant virions selectively bound DC receptors to allow DC-specific membrane fusion. This nanobody technology has proven successful at targeting lentiviral vectors to murine DCs both in vitro and in situ and has previously been discussed [75,76]. Further, because of their ability to mediate host effector and memory CD8+ T cell responses, which is crucial for anti-tumor immunity, DC-based vaccines have become one of the leading strategies for cancer immunotherapy [77]. However, in the case of therapeutic HIV vaccines, vector-transduced DCs can themselves act as latency-reversing agents (LRAs) by secreting high levels of cytokines, such as TNF-α, and they are also capable of homing to lymphoid tissues where latently-infected viral reservoirs reside to potentially activate latent viral reservoirs. On the other hand, concerns related to DC-based vaccines are: (i) DCs could induce inflammation in the tissues and (ii) monocyte-derived DCs are susceptible to tumor-mediated immunosuppression although this is not important in the case of lentiviral-based HIV DNA vaccines.

## 4. Large-Scale Production of Lentiviral Vectors

Large-scale production of viral vectors initiates with the generation/availability of a sufficiently large number of packaging cells or producer cells, usually HEK293T cells. Subsequently, packaging cells are transiently transfected with vectored DNA encoding necessary proteins for lentiviral vector production. In order to enhance the infectivity of viral particles to a wide range of cells, pseudotyped, HIV-based lentiviral particles are produced. The core packaging plasmids include envelope protein, usually vesicular stomatitis virus protein G (VSV-G), HIV-1 Gag and Pol genes, and HIV accessory proteins, such as Rev. Later, packaging cells, such as HEK293T cells are co-transfected with these constructs. After 48 h of transfection, the lentiviral particles are obtained by collecting the culture medium of the packaging cells. After removal of cell debris by filtering the supernatant, the viral particles are treated to remove contaminating DNA products. Subsequently, lentiviral particles are purified using different methods, which include gradient purification or chromatography. Once purified, the eluted fractions undergo a series of filtration steps to sterilize and remove any remaining cellular debris [78,79].

The purity of the product is critical because the debris from the packaging cells can easily contaminate the vector product, and these impurities may cause inflammation during in vitro and in vivo studies [80]. Once prepared, the lentiviral vector stocks can remain viable and stable for up to 9 years following cryopreservation at −80 °C [81].

## 5. Safety Considerations of Lentiviral Vectors

The lentiviral vectors belonging to earlier generations consisted of a large portion of the HIV genome, including the Gag and Pol genes, besides several additional viral proteins [24]. In order to induce the target population beyond CD4+ cells, the lentiviral particles are pseudotyped with the envelope protein of another virus, usually VSV-G. The VSV-G binds to a ubiquitously expressed cell surface receptor that has been identified as the low-density lipoprotein (LDL) receptor [82,83]. This allows the VSV-G pseudotyped lentiviral vector to transduce a vast range of cells [84]. Given that higher levels of VSV-G are toxic to the cell, the VSV-G gene is expressed through a separate plasmid. The first-generation of lentiviral vectors consists of almost all HIV genes, including accessory genes Vif, Vpr, Vpu, and Nef, as well as the regulatory genes, Tat and Rev. The Vif, Vpr, Vpu, and Nef provide survival advantages for lentiviral replication in vivo, although they are dispensable for the growth of the virus in vitro. Tat and Rev are required for viral replication. However, in the second generation of lentiviral vectors, accessory factors Vif, Vpr, Vpu, and Nef were removed. This modification substantially reduced the virulency and toxicity of lentiviral particles [24]. Notably, the removal of the accessory genes did not affect the transfer of genetic material to the host cells. Subsequently, to further improve the safety of lentiviral vectors in the third-generation of lentiviral vectors, the Gag and Pol genes were encoded on a different construct from that of the Rev or Env genes. Thus, four separate constructs are required to generate the third-generation lentiviral particles in the packaging cell line. The four constructs include; packaging, transfer, envelope, and Rev-expressing constructs. This modification greatly enhances the safety by splitting the viral genome into separate constraucts, making recombinant virus generation almost impossible [85]. Moreover, in third-generation lentivectors, Tat gene was also removed because a constitutively active promoter was inserted upstream of the long terminal repeat (LTR) to express HIV sequences. In order to further improve safety, deletions into the 3’LTR of the viral genome were created to enable self-inactivating (SIN) lentiviral vectors to disrupt the promoter/enhancer activity of the LTR [85]. The choice of internal promoters used in the third-generation SIN lentiviral vectors is important. Initial studies, which used the cytomegalovirus immediate early gene promoter, also showed robust expression in most cell lines that are actively dividing. However, in primary cells, such as the CD34+ stem cells and T-cells, promoters vary substantially in their activity [86], with the cytomegalovirus promoter showing greater variation with T-cell activation than with constitutively active cellular promoters, such as human elongation factor-1 alpha (EF-1α) [87]. Genetic alterations and modifications of lentiviral vectors have enabled improved safety and efficacy, reduced the administration dose, and also enabled efficient large-scale production for vaccine development and other applications such as gene therapy.

Nonetheless, there are several concerns with lentiviral vectors that need to be addressed, especially safety. Safety concerns include the possibility of reverting into replication-competent species, insertional mutagenesis as a result of integration into the genome of the vaccinated individuals, and vector mobilization. Another safety concern is the possible development of autoimmunity as well as antibiotic resistance due to the presence of antibiotic resistance genes contained within the lentiviral construct. Isaguliants et al. [88] immunized eleven mice with DNA/protein vaccines, out of which seven developed secondary antibodies against DNA at fifty weeks from the start of immunization. Precautions have been taken to address some of these safety issues with lentiviral vectors. For instance, the split genome design is intended to prevent the formation of replication-competent species in vaccinated individuals. In addition, all sequences that encode retroviral proteins are deleted from the vector, leaving only those that are required for efficient packaging into viral particles [89]. Furthermore, the issue of vector mobilization is addressed by the deletion of the enhancer/promoter sequences from the 3′ long terminal repeat (LTR). Despite all of these efforts to improve the safety of lentiviral vectors, many of the safety problems remain largely unaddressed.

## 6. Vaccination Approaches Demonstrated to Enhance Immune Responses

Pre-clinical studies have shown that lentiviral vectors induce strong HIV-specific adaptive immune responses [48,49,54,55,56,57,90]. Lentiviral vectors expressing HIV-1 or SIV surface proteins, in both mouse models and human in vitro studies, have been shown to induce strong HIV-specific humoral and cytotoxic T-lymphocytes (CTLs) [49,54,56,57,90]. Interestingly, it was noted that an HIV-1-based lentiviral vector encoding HIV Gag, Pol, and Rev (VRX1023) induces more potent and durable mucosal and systemic cellular and humoral immune responses compared with adenovirus-based vectors [57]. A single dose of the lentiviral vector elicited strong and diverse Gag-specific T-cell responses, which peaked 16 days following prime-boost regardless of the dose used. However, a subsequent challenge with high-dose SIVmac251 resulted in an infection in all animals although the acute phase of infection demonstrated a more than two-fold reduction in viral replication and protection from CD4+ T-cell depletion.

Most recently, Joachim et al. [91] evaluated antibody responses to the HIV envelope variable region in twenty-nine individuals who received HIV DNA prime and HIV-MVA boost in phase I and II clinical trials. They observed that HIV DNA/MVA vaccine regiment induced durable V1V2 immunoglobulin G (IgG) antibody responses in the majority of the vaccinated individuals. Similarly, Msafiri et al. [92] also reported frequent antibody responses directed at the V1V2 region of the glycoprotein 120 induced by HIV DNA prime followed by HIV-MVA boost in healthy African volunteers. Furthermore, several recent clinical trials evaluated DNA vaccine delivery strategies that enhanced the expression of heterologous antigens and improved immune stimulation. In this regard, in a randomized placebo-controlled trial of HIV DNA prime and HIV MVA boost vaccination, Nilsson et al. [37] compared the safety and immunogenicity of intradermal (ID) vaccination with or without electroporation (EP) in healthy Swedish volunteers. They observed that, although ID/EP of HIV DNA was well tolerated, strong cell- and antibody-mediated immune responses were elicited by HIV DNA prime and HIV MVA boost vaccination with or without ID/EP. In a related development, in a randomized trial in Mozambique, Viegas et al. [93] evaluated ID HIV DNA immunization using needle-free Zetajet injection followed by HIV-MVA boost and found it to be safe and immunogenic. Similarly, Bakari et al. [94] reported broad and potent immune responses to a low-dose ID HIV DNA prime, boosted with recombinant HIV-MVA among healthy adults in Tanzania. Likewise, Hossenipour et al. [95] most recently compared the safety and immunogenicity of DNA prime followed by DNA/protein boost. The DNA/protein boost was co-administered intramuscularly (IM) via needle or needle-free injection device (Biojector). They observed that all vaccinations were safe and well tolerated. Further, they observed that DNA/protein co-administration was associated with HIV-1 V1V2 antibody responses. However, DNA administration by Biojector elicited higher CD4+ T cell responses to HIV envelope protein compared with the needle injection.

## 7. Integrase-Defective Lentiviral Vectors

Unlike integrating lentiviral vectors, IDLVs have improved safety profiles. IDLVs solve the safety issues associated with integrating lentiviral vectors. However, transgene retention remains a problem since episomes are rapidly diluted out through cell division. Nonetheless, Kymalainen et al. [96] developed an IDLV system that generated mitotically stable episomes with the capacity for long-term transgene expression. Verghese et al. [97] also developed a novel approach that enabled long-term mitotic maintenance of IDLV episomes. Furthermore, Negri et al. [98] evaluated the immunogenicity of an SIV-based IDLV in a non-human primate. In this case, six rhesus monkeys were intramuscularly primed with IDLV-ENV and also boosted with the same vector a year later. They reported that a single immunization with IDLV-ENV elicit broad cellular and humoral immune responses, which although waned over time, were still detectable after one-year post-prime. However, the boost with IDLV-ENV after a year induced a remarkable increase in both humoral and T cell responses. Furthermore, Blasi et al. [99] evaluated the immunogenicity, safety, and efficacy of sequential immunization with an SIV-based IDLV in rhesus macaques. They observed that immunization with IDLV expressing sequential CH505 ENVs induced a highly long-lasting and strong and neutralizing antibody response compared with protein or DNA plus protein immunization with the same sequential envelopes. Besides, there was no evidence of vector mobilization or recombination in the immunized and subsequently challenged monkeys suggesting the potential use of IDLVs for prophylactic HIV vaccines. Similar results were also reported by Gallinaro et al. [100].

Although highly active antiretroviral therapy (HAART) can control HIV replication and prevent viral transmission, it is unable to eradicate HIV proviral reservoirs which are capable of reactivating productive lytic infection following interruption of HAART, suggesting the failure of the host immune responses to control viral replication in cellular reservoirs of latent HIV. Therefore, in addition to potential use of IDLVs for prophylactic HIV vaccine, therapeutic HIV vaccine is one of the approaches currently being considered to improve antiviral host immune responses to enable long-term viremia control. Consistent with this approach, using IDLV, Blasi et al. [101] demonstrated that expressing SIV-Gag to boost anti-Gag specific immune responses, a single immunization with IDLV-Gag induced durable viral control in 55% of the vaccinated macaques, which correlated with an increase in SIV-Gag specific CD8+ T cell responses. Similarly, Nakamura-Hoshi et al. [102] demonstrated that therapeutic vaccine induced Gag-specific CD8+ T cells with augmented anti-virus efficacy in simian immunodeficiency virus-infected macaques under HAART.

## Figures and Tables

**Table 1:** Advantages and disadvantages of major viral vectors for DNA vaccine delivery.

Sl.no	Virus	Advantages	Disadvantages	Reference
1.	Lentivirus	Can infect non-dividing cells, long-term gene expression, and can generate high immunogenicity	Chance of generation of replication-competent virus and potential for tumorigenesis	[26,27]
2	Adenovirus	Safety and high titer production	Pre-existing immunity	[28]
3	Adeno-associated virus	Long-term gene expression, non-pathogenic virus, and induces a unique CTL response	Low titer production and pre-existing immunity	[29]
4	Retrovirus	Long-term gene expression	Chance of generation of replication-competent virus, potential for tumorigenesis, and infects dividing cells only	[30]
5	Vaccinia virus	Safety, high titer production, and can generate high immunogenicity	Pre-existing immunity	[31]
6	Cytomegalovirus	Protects against SIV infection	Risk of pathogenesis in specific individuals	[32]
7	Sendai virus	Can generate high immunogenicity	Pre-existing immunity	[33]

## Data Availability

Not applicable.
